# Protective effect of phytoestrogens on nonalcoholic fatty liver disease in postmenopausal women

**DOI:** 10.3389/fphar.2023.1237845

**Published:** 2023-08-30

**Authors:** ChenLu Zhao, JunHao Shi, DongFang Shang, Min Guo, Cheng Zhou, WenXia Zhao

**Affiliations:** ^1^ First Clinical Medical College, Henan University of Traditional Chinese Medicine, Zhengzhou, China; ^2^ Department of Digestive Diseases, The First Affiliated Hospital of Henan University of Traditional Chinese Medicine, Zhengzhou, China

**Keywords:** phytoestrogens(PEs), postmenopausal women, non-alcoholic fatty liver disease, lipid metabolism, glucose metabolism, oxidative stress, inflammatory response, intestinal flora

## Abstract

Non-alcoholic fatty liver disease (NAFLD) is a progressive metabolic disease characterized by hepatic steatosis, inflammation, and fibrosis that seriously endangers global public health. Epidemiological studies have shown that the incidence of non-alcoholic fatty liver disease in postmenopausal women has significantly increased. Studies have shown that estrogen deficiency is the main reason for this situation, and supplementing estrogen has become a new direction for preventing the occurrence of postmenopausal fatty liver. However, although classical estrogen replacement therapy can reduce the incidence of postmenopausal NAFLD, it has the risk of increasing stroke and cardiovascular diseases, so it is not suitable for the treatment of postmenopausal NAFLD. More and more recent studies have provided evidence that phytoestrogens are a promising method for the treatment of postmenopausal NAFLD. However, the mechanism of phytoestrogens in preventing and treating postmenopausal NAFLD is still unclear. This paper summarizes the clinical and basic research evidence of phytoestrogens and reviews the potential therapeutic effects of phytoestrogens in postmenopausal NAFLD from six angles: enhancing lipid metabolism in liver and adipose tissue, enhancing glucose metabolism, reducing oxidative stress, reducing the inflammatory response, regulating intestinal flora, and blocking liver fibrosis (Graphical Abstract).

## Graphical Abstract

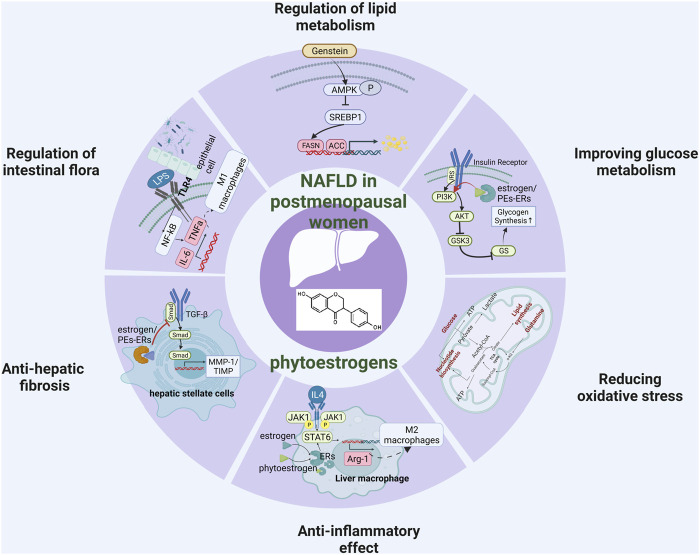

## Introduction

Non-alcoholic fatty liver disease (NAFLD) is a major health problem with rising incidence of obesity and diabetes in many countries, and the prevalence rate is as high as 25% in the general population (Cotter and Rinella, 2020). NAFLD is a progressive liver disease with a wide spectrum of diseases. It begins with simple steatosis, can progress to nonalcoholic steatohepatitis (NASH), and even further develop into liver fibrosis or hepatocellular carcinoma (HCC) (Sheka et al., 2020). Epidemiological data have indicated a higher rate of NAFLD in postmenopausal women. For example, in some clinical investigations, the prevalence of NAFLD was lower in premenopausal women than in men (12.7% vs. 26%), but considerably higher in postmenopausal women than in males of the same age (19.4% vs. 14.9%) (Lonardo et al., 2019; DiStefano, 2020). Furthermore, Trembling et al. and Sumida et al. confirmed that long-term estrogen deficiency may increase the risk of NAFLD fibrosis in postmenopausal women (Sumida et al., 2020; Trembling et al., 2020). Similarly, the ovariectomized (OVX) rodent models suggest a causal relationship between estrogen deficiency and increased susceptibility to NAFLD (Li et al., 2013; Jeong et al., 2018; Chang et al., 2020). Estrogen deficiency plays an important role in NAFLD pathogenesis in postmenopausal women. Several studies have reported the benefits of estrogen replacement therapy (ERT), reducing the prevalence of postmenopausal NAFLD (Yang et al., 2017; Polyzos et al., 2022). In contrast, some investigators have proposed that ERT may increase the risk of cardiovascular disease, stroke, and breast cancer (Santen et al., 2020; Yoshida et al., 2022). Plant-derived phytoestrogens have a similar chemical structure and biological activity to human estrogens. Phytoestrogens have weak estrogenic activity by binding to estrogen receptors α or β (Sirotkin and Harrath, 2014). In this review, we systematically summarize the potential mechanisms of phytoestrogens in postmenopausal NAFLD, such as improving the lipid metabolism in the liver and adipose tissue, improving glucose metabolism, alleviating oxidative stress, reducing the inflammatory response, regulating intestinal microbiota, and stemming liver fibrosis.

### A brief introduction of phytoestrogens

**FIGURE 1:**
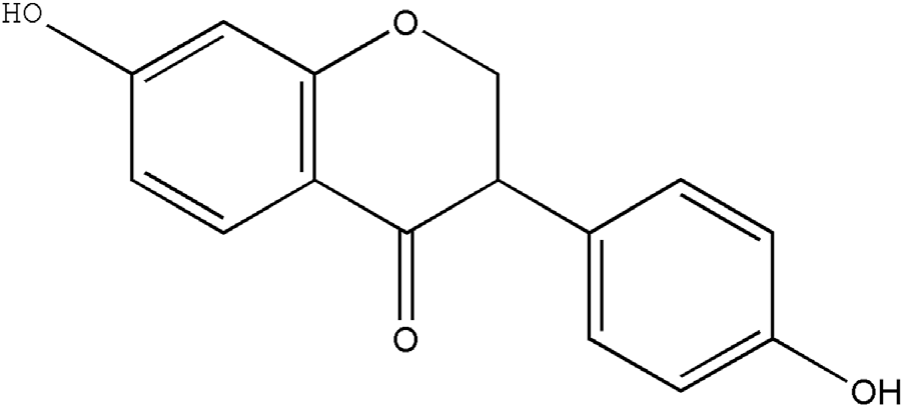
Molecular structure of phytoestrogens.

Phytoestrogens are a class of heterocyclic polyphenols existing in plants whose composition includes two hydroxyl groups and one phenolic ring (Figure 1). The phenolic ring controls how tightly phytoestrogens bind to receptors. Because phytoestrogens function as both estrogen agonists and antagonists, they are often referred to as selective estrogen receptor modulators (SERMs) (Brzezinski and Debi, 1999). As an estrogen agonist, phytoestrogens can combine with estrogen receptor (ER) to play a weak estrogen effect (Rietjens et al., 2017). As estrogen antagonists, they can block estrogen receptors and inhibit estrogen activity, causing anti-estrogen effect. The bioavailability of phytoestrogens depends on the form of action, dosage, individual metabolism, other drug intake factors, target tissue concentration dependence, and whether endogenous estrogen exists or not (Joannou et al., 1995; Xu et al., 1995; Wiseman, 1999; Glazier and Bowman, 2001).

The reported phytoestrogens include isoflavones, coumarins, lignans, anthracenes, chalcones, and saponins (Matsuda et al., 2001; Chan et al., 2002; Rietjens et al., 2017). According to the structure of phytoestrogens, they are mainly divided into flavonoids, stilbenes, lignans, coumarins, and mycoestrogens. (i) Flavonoids, such as daidzein, genistein, calycosin, and so on, mainly exist in Leguminosae. They usually exist in the conjugate forms of genistein, daidzein, puerarin, daidzein, sandalwoodin, and sissotrin (Rietjens et al., 2017). (ii) Stilbenes, such as 6-isoprenyl naringenin, 6-vanillyl naringenin, 8-isoprenyl naringenin and isoflavones, 8-isoprenyl naringenin is the phytoestrogen with the most obvious estrogenic effect. They mainly exist in higher plants such as pine, mulberry, Gnetaceae, Cyperaceae, Fabaceae, Dipterocarpaceae, and Vitaceae (Tanwar et al., 2021). (iii) Lignans, including intestinal diols and enterolactones, are converted from lignan precursors under the action of intestinal flora (Lampe, 2003). Other intestinal lipid precursors identified include Arctigenin, 7-hydroxy lycopene, lariciresinol, pinoresinol and syringaresinol (Meagher et al., 1999; Heinonen et al., 2001). Lignans are abundant in flaxseed, whole wheat bread, fruits, vegetables, sesame, tea and other foods (Rietjens et al., 2017; Tanwar et al., 2021). (iv) Coumarins include psoralen, coumarins, 4' -methoxycoumarin, angelica visfatin, repensol, trifoliate phenol, etc. They mainly exist in legumes, especially in edible plants, such as peas, mung bean sprouts, alfalfa, clover sprouts (Rietjens et al., 2017). (v) Mycoestrogens, a natural estrogen produced by fungi, is harmful to animals when eating contaminated feed. Zearalenone (ZEA) is a fungal estrogen that has been studied extensively. ZEA is widely found in contaminated foods. Due to its strong estrogenic activity, ZEA is considered to be a reason for female reproductive changes (Xu et al., 2018).

### Regulation of lipid metabolism in liver

**FIGURE 2:**
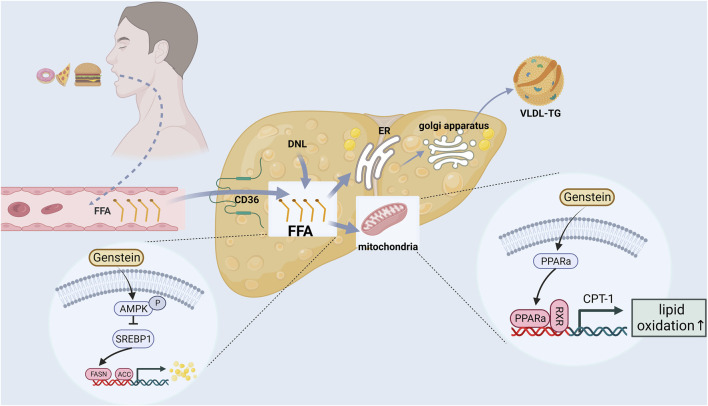
The mechanism of phytoestrogens regulating liver lipid metabolism.

Liver lipid metabolism includes lipid uptake and production, output and oxidation. Breaking one or more balance can promotes liver steatosis (Jones, 2016; Ipsen et al., 2018). In addition, postmenopausal women are prone to systemic fat redistribution due to estrogen deficiency. The risk of abdominal obesity in postmenopausal women is significantly higher than that in premenopausal women (5 times) (Donato et al., 2006). Therefore, restoring the lipid metabolism balance is a crucial link in the prevention and treatment of postmenopausal NAFLD (Figure 2).

Genistein is the most abundant phytoestrogen in soybean and is one of the most studied phytoestrogens. Several studies have found that genstein can reduce the body weight, liver weight, serum and liver lipid levels (triglyceride, cholesterol, serum free fatty acids) of ApoE (−/−) (Jeon et al., 2014), C57BL6J mice (Kim et al., 2010), Sprague-Dawley (SD) rats (Liu H. et al., 2017) and SD-ovariectomized rats (Witayavanitkul et al., 2020) by the following mechanisms: (i) Reducing free fatty acid (FFA) intake and liver lipid source (Jeon et al., 2014). Genstein reduced hepatic TG and FFA by inhibiting scavenger receptor, CD36 and scavenger receptor A uptake by oxidized low density lipoprotein. (ii) Inhibition of lipid synthesis related gene expression (Liu H. et al., 2017; Seidemann et al., 2021). On the one hand, genstein could downregulate the expression of AMP-activated protein kinase/acetyl-coenzyme A carboxylase (AMPK/ACC) signaling pathway, inhibited the expression of downstream fatty acid synthase and 3-phosphate glyceryl transferase (GPAT), and reduced liver lipid synthesis. On the other hand, Genstein inhibited the expression of sterol regulatory element binding protein 1 (SREBP1), thereby reducing the hepatic *de novo* adipogenesis (DNL) pathway. (iii) Reducing lipid peroxidation and promoting gene expression related to FFA oxidative decomposition (Kim et al., 2010; Zhong et al., 2017; Seidemann et al., 2021). Genstein promoted the expression of peroxisome proliferator-activated receptor α (PPARα), carnitine palmitoyl transferase-1 (CPT-1) and acyl-CoA oxidase (ACO), thereby accelerating the β-oxidation of FFA, and ultimately reducing liver lipid accumulation and lipid toxicity. Amanat et al. (Amanat et al., 2018) randomly divided 82 NAFLD subjects into treatment group and placebo group. The treatment group was supplemented with 250 mg genistein daily for 8 weeks. At the end of the experiment, it was found that the waist-to-hip ratio, body fat percentage and TG of NAFLD subjects supplemented with genistein were significantly lower than those of the placebo group. Another cross-sectional study of 6786 adults in China (Wang et al., 2022) found that dietary isoflavone intake was negatively correlated with the prevalence of NAFLD, hyperlipidemia and hypertension.

### Regulation of lipid metabolism in adipose tissue

In Ji-Hye Jung study (Jung and Kim, 2013), mice were fed with a high cholesterol/fat diet (HCD) and treated with different concentrations of black soybean powder, showing different effects on improving liver and adipose tissue lipid metabolism. The levels of total cholesterol (TC) and TG and the expression of SREBP2 in the liver of mice treated with 4% black soybean powder were significantly lower than those in the model group. Black soybean powder could stimulate the secretion of adiponectin, activate the expression of pAMPK and eliminate FFA in the liver. Similarly, daidzein treatment (Cao et al., 2013) increased serum adiponectin levels and decreased body weight gain, visceral fat gain and HOMA-IR index in OVX rats. Naoki Nanashima et al. (Nanashima et al., 2020) used 3% blackcurrant to treat OVX rats for 3 months. At the end of the experiment, it was found that 3% blackcurrant significantly reduced the body weight, visceral fat weight, TG, TC and low-density lipoprotein (LDL) of rats. More importantly, hematoxylin and eosin staining (HE) showed that blackcurrant (Ribes nigrum L.) extract (BCE) reduced the diameter of adipocytes and the score of NAFLD activity. C57BL/6J mice fed with genistein (Kim et al., 2010) showed a dose-dependent decrease in body weight and lipid levels. Genistein also inhibited adipocyte hypertrophy and adipogenesis by down-regulating the expression of LXRα, SREBP1c and PPARγ. In Narrerat study (Sutjarit et al., 2018), it also was found that comosa Roxb could inhibit adipocyte size and regulate adipokine secretion in OVX rats. Panneerselvam et al. (2016)found that soy isoflavones (150 mg/kg body weight/day, 8 weeks) improved hepatic steatosis in HFD-fed ovariectomized Wistar rats by down-regulating insulin-inducible gene 2 (insig2) and PPARα expression in adipose tissue, inhibiting adipocyte differentiation and reducing fat formation. Kim et al. (2020) found that coumarin (100 μg/20g body weight/day, 2 weeks) can activate brown adipose tissue (BAT) in HFD-fed C57BL/6J mice, increase BAT mitochondria, and accelerate BAT energy metabolism. The Shen et al. (2019); Kang et al. (2020)have proved that Genstein and Secoisolariciresinol diglucoside (SDG) can upregulate the expression of peroxisome proliferator-activated receptor gamma coactivator 1α (PGC1α), promote white fat browning, and increase body heat production and reduce fat accumulation through AMPK pathway. These basic studies provide evidence for phytoestrogens to prevent lipid metabolism disorders and NAFLD in postmenopausal women. Table 1 lists other studies on phytoestrogens improving lipid metabolism in ovariectomized models (Dai et al., 2004; Böttner et al., 2008; Pakalapati et al., 2009; Miller et al., 2015; Cross et al., 2017; Chen et al., 2018; Zheng et al., 2018; Zingue et al., 2018; Huang et al., 2020; Tang et al., 2021).

**TABLE 1:** Effects of phytoestrogens on lipid metabolism in ovariectomized models.

Phytoestrogen	Models	Period	Dosage	Main results	Ref
Bazi Bushen capsule (BZBS)	HFD-fed ovariectomized C57BL/6J ApoE^−/−^ mice	12 weeks	1.4/2.8 g/kg	1.Serum TG↓, TC↓,LDL↓,HDL↑	Huang et al. (2020)
				2.Serum DHA↑, Lysophosphatidylethano-lamine ↓	
C.athayensis (CCE)	HFD-fed ovariectomized SD rats	8 weeks	50/100/200 mg/kg	1.Body weight↓, abdominal fat coefficients↓	Tang et al. (2021)
				2.Serum FFA↓, TG↓, TC↓, lipid droplets in liver cells↓	
				3.Serum leptin↓, adiponectin↓	
				4.Diameter of abdominal fat cells↓	
Zearalanol (ZEN)	Cholesterol-fed ovariectomized rabbits	12 weeks	0.1/0.5/2.5 mg/kg	1.Serum TC↓, TG↓,HDL↑, LDL↓	Dai et al. (2004)
				2.ApoA1↓,ApoB↓	
Soy	Ovariectomized LCR rats	28 weeks	585 mg/kg	1.Body weight↓, fat↓, WAT weight↓	Cross et al. (2017)
				2.Hepatic TG↓	
				3.Insulin sensitivity↑	
				4.Adipose tissue mRNA levels:CD11c↓,IL6↓,TNFα↓	
				5.Abundance of Firmicutes↓, abundance of Bacteroidetes↑, F/B↓	
8preny -lnaringenin (8-PN)	Ovariectomized rats	3 months	6.8/68.4 mg/kg	1.Body weight↓	Böttner et al. (2008)
				2.Serum TC↓, LDL↓, HDL↑	
equol/isoliquiritigenin/glabridin/genistein	Ovariectomized SD rats/LO2 cells	50 days	Equol:10 mg/kg; isoliquiritigenin/glabridin/genistein:50 mg/kg	1.Body weight↓	Chen et al. (2018)
				2.Blood Glucose↓, Hepatic TG↓	
				3.Gene expression related to lipid metabolism: SREBP1↓, ACC1↓, FAS↓,SCD1↓	
				4.HE staining: liver steatosis↓	
Mixture:genistein(G)/resveratrol (R)/quercetin(Q)	AIN-93M-fed ovariectomized Fischer rats	16 weeks	Diet1:1000 mg/kg (G); Diet2:500 Mg/kg(G)+200 mg/kg(R)+1000 mg/kg(Q); Diet3:1000 mg/kg(G)+400 mg/kg(R)+2000 mg/kg(Q)	1.All three diets: retroperitoneal lipid content ↓	(Miller et al., 2015)
				2.All three diets: blood Glucose↓	
				3.Diet1: Serum FFA↓,ALT↓, DGAT1↓	
				4.Diet1 and Diet3:SCD1↓,XBP1↓	
Millettia macrophylla	Ovariectomized Wistar rats	28 days	10 mg/kg	1.Body weight↓, abdominal fat↓	Zingue et al. (2018)
				2.Dyslipidemia↓, glucose intolerance↓	
Isoflavone and exercise	Ovariectomized Wistar rats	61 days	4 mg/kg	1.visceral fat mass↓, adipocyte size↓	Zheng et al. (2018)
				2.Serum leptin↓	
				3.SREBP-1c↓,FAS↓,PPARδ↑,PGC-1α↑	
Trifolium pratense	Ovariectomized SD rats	4 days	450 mg/kg	1.Serum TC↓, LDL↓, HDL↑	Pakalapati et al. (2009)
				2.Changes in protein-coding genes expression in lipid metabolism, antioxidant and xenobiotic metabolism	

↑: Increased; ↓: Decrease.

BZBS, bazi bushen capsule; TG, triglyceride; TC, cholesterol; LDL, low density lipoprotein; HDL, high density lipoprotein; DHA, docasa-hexaenoic-acid; CCE, C. athayensis; SD, Sprague-Dawley; FFA, free fatty acid; ZEN, zearalanol; ApoA1, serum apolipoprotein a1; ApoB, serum apolipoprotein B; WAT, white adipose tissue; CD11c, a specific marker of M1 macrophages; IL6, interleukin-6; TNFα, tumor necrosis factor-α; F/B, M. intestinalis/*Bacteroides*; 8-PN, 8prenylnaringenin; SREBP1, sterol regulatory element binding protein 1; ACC1, acetyl coenzyme A carboxylase 1; FAS, fatty acid synthetase; SCD1, stearoyl-coenzyme a desaturase 1; HE, hematoxylin and eosin staining; G, Mixture:genistein; R, resveratrol; Q, quercetin; ALT, alanine transaminase; DGAT1, Diacylglycerol O-acyltransferase 1; XBP1, X-box binding protein 1; PPARδ, peroxisome proliferator-activated receptor δ; PGC-1α, Peroxisome proliferator-activated receptor γ coactivator-1α.

### Improving glucose metabolism

Insulin resistance (IR) is a decline in insulin sensitivity or responsiveness in target organs (such as liver, adipose tissue, skeletal muscle, etc.) (Lebovitz, 2001). IR is the key link in the occurrence of NAFLD. As the initiating factor of “first hit”, IR is closely related to “second hit” factors such as lipid peroxidation, oxidative stress and inflammatory response (Day, 2002; Fang et al., 2018; Bessone et al., 2019). Due to estrogen deficiency in postmenopausal women, the expression of insulin receptor and insulin receptor substrate-1/2 (IRS-1/2) is reduced, and the insulin signal transduction is weakened, resulting in IR and eventually inducing NAFLD (Mauvais-Jarvis et al., 2017).

Genistein could reduce the serum insulin level in NASH model of SD rats and improve HOMA-IR in a dose-dependent manner (Yin et al., 2019). Similarly, in the OVX model of SD rats, supplementation of daidzein (50 mg/kg body weight/day, 12 weeks) could also reduce body weight, HOMA-IR and fasting insulin level (Cao et al., 2013). Another study showed that genistein alone or in combination with metformin significantly reduced fasting blood glucose (FBS) in HFD mice by reducing glucose 6-phosphatase (G6Pase) and increasing glycogen synthase kinase 3β (GSK-3β) phosphorylation, thereby inhibiting gluconeogenesis (Zamani-Garmsiri et al., 2021). *In vitro* studies by Tomasz et al. (Charytoniuk et al., 2019) found that enterolactone could reduce the phosphorylation levels of Serine/threonine kinases (AKT) and AMPK in HepG2 cells induced by palmitic acid, and ultimately improve liver insulin sensitivity.

In addition, clinical studies have yielded encouraging findings. In a randomized double-blind controlled trial (Amanat et al., 2018), compared with placebo, genistein supplementation (250 mg/day, 8 weeks) could reduce insulin levels and HOMA-IR in NAFLD patients. In another randomized double-blind control experiment involving 54 postmenopausal patients with type 2 diabetes, compared with placebo, genistein supplementation (108 mg/day, 12 weeks) could significantly reduce the subjects’ FBS, glycosylated hemoglobin, TG and malondialdehyde (MDA) (Braxas et al., 2019). However, the dose and time of genistein used in these two studies are different, which may be related to different dosage forms and subjects of genistein. A meta-analysis (Liu Y. et al., 2017) concluded that genistein significantly improved blood glucose levels and insulin sensitivity in postmenopausal women and that long-term treatment may be more effective than short-term use. Therefore, more clinical trials are still needed to determine the optimal dose and course of treatment of phytoestrogens for different diseases.

### Reducing oxidative stress

Oxidative stress occurs when the production of oxidative molecules (such as superoxide, hydrogen peroxide) exceeds the scavenging capacity of antioxidant molecules (such as catalase (CAT), glutathione peroxidase (GSH-Px), superoxide dismutase (SOD)) (Sies, 2015). When oxidation and antioxidant imbalance occurs, it will cause lipid and protein peroxidation, nucleic acid oxidative modification, and other metabolic diseases such as NAFLD.

Phytoestrogens have been proved to have antioxidant effects in various models. In OVX-NASH rat model, compared with OVX model group, genistein supplementation could significantly reduce liver MDA and increase reduced glutathione (GSH) levels (Witayavanitkul et al., 2020). In OVX-LDLR (−/−) atherosclerosis model mice and HAEC cell model, dioscin could reduce the levels of MDA and reactive oxygen species (ROS), and increase the levels of GSH and nicotinamide adenine dinucleotide phosphate oxidase 4 (NOX4), which was related to the activation of PGC-1α/ERα pathway (Yang et al., 2019). In the aging rat model, compared with the control group, the supplementation of Fructus Corni extract increased liver SOD, CAT and GPX, and decreased liver lipid peroxidation, suggesting that Fructus Corni extract may be a ROS scavenger (Hamden et al., 2009). Similarly, in the Granulosa cells model, genistein significantly increased mitochondrial membrane potential and enhanced the expression of SOD, GPX, CAT and adenosine-3′, 5′-cyclic monophosphate (cAMP), which was related to cAMP-PKA signaling pathway (Luo et al., 2020). In clinical studies, it is also found that phytoestrogens have antioxidant effects. Compared with placebo, genistein supplementation could reduce the serum MDA levels in NAFLD patients and postmenopausal type 2 diabetes (T2DM) patients (Amanat et al., 2018; Braxas et al., 2019). In summary, phytoestrogens maintain the balance of oxidation/antioxidant system directly or indirectly to achieve antioxidant effect *in vivo* and *in vitro* models. Table 2 summarizes the basic research of other phytoestrogens on improving oxidative stress in ovariectomized models (Baeza et al., 2010; Chulikhit et al., 2021; Jdidi et al., 2021; Ltaif et al., 2021).

**TABLE 2:** Effects of phytoestrogens on oxidative stress in ovariectomized models.

Phytoestrogen	Models	Period	Dosage	Main results	Ref
A. sativa	Ovariectomized Swiss mice	60 days	200 mg/kg	1.Serum TG↓,VLDL↓,ALT↓	Ltaif et al. (2021)
				AST↓,ALP↓, LDH↓	
				2.Hepatic AOPP↓	
				3.Hepatic GPx↓, GSH↓, SOD↑	
Medicago sativa	Ovariectomized white Swiss mice	8 weeks	0.75 g/kg	1.Serum TG↓,TC↓,HDL↑	Jdidi et al. (2021)
				2.Hepatic GPX↓,GSH↓	
				SOD↑	
soybean	aged ovariectomized Wistar rats	10 weeks	300 mg/kg	1.liver, heart, kidney, spleen homogenates: GSSG/GSH↓, MDA↓	Baeza et al. (2010)
Pueraria mirifica (PM)	Ovariectomized mice	8 weeks	2.5/25 mg/kg	1.CAT↑,SOD↑,IL6↓,TNF-α↓	Chulikhit et al. (2021)

↑: Increased; ↓: Decrease.

VLDL, very low density lipoprotein; AST, glutamic-oxalacetic transaminase; ALP, alkaline phosphatase; LDH, lactic dehydrogenase; AOPP, advanced oxidation protein products; GPx, glutathione peroxidase; GSH, glutathione; SOD, superoxide dismutase; GSSG/GSH, oxidized glutathione/glutathione; MDA, malondialdehyde; PM, pueraria mirifica; CAT, catalase.

### Anti-inflammatory effect

Estrogen at physiological concentration can inhibit the release of tumor necrosis factor α (TNFα), interleukin-6 (IL-6) and interleukin-1β (IL-1β). A cross-sectional study (Rodrigues et al., 2014) found that serum IL-6 and TNFα levels in postmenopausal NAFLD patients with metabolic syndrome were higher than those in the control group, suggesting that estrogen deficiency would promote or aggravate the development of NAFLD. Phytoestrogens have a good inhibitory effect on liver inflammation in NAFLD. In patients with NAFLD, Amanat’s study (Amanat et al., 2018) demonstrated that genistein 250 mg daily for 8 weeks could lower TNFα and IL-6 levels and alleviate liver inflammation.

**FIGURE 3:**
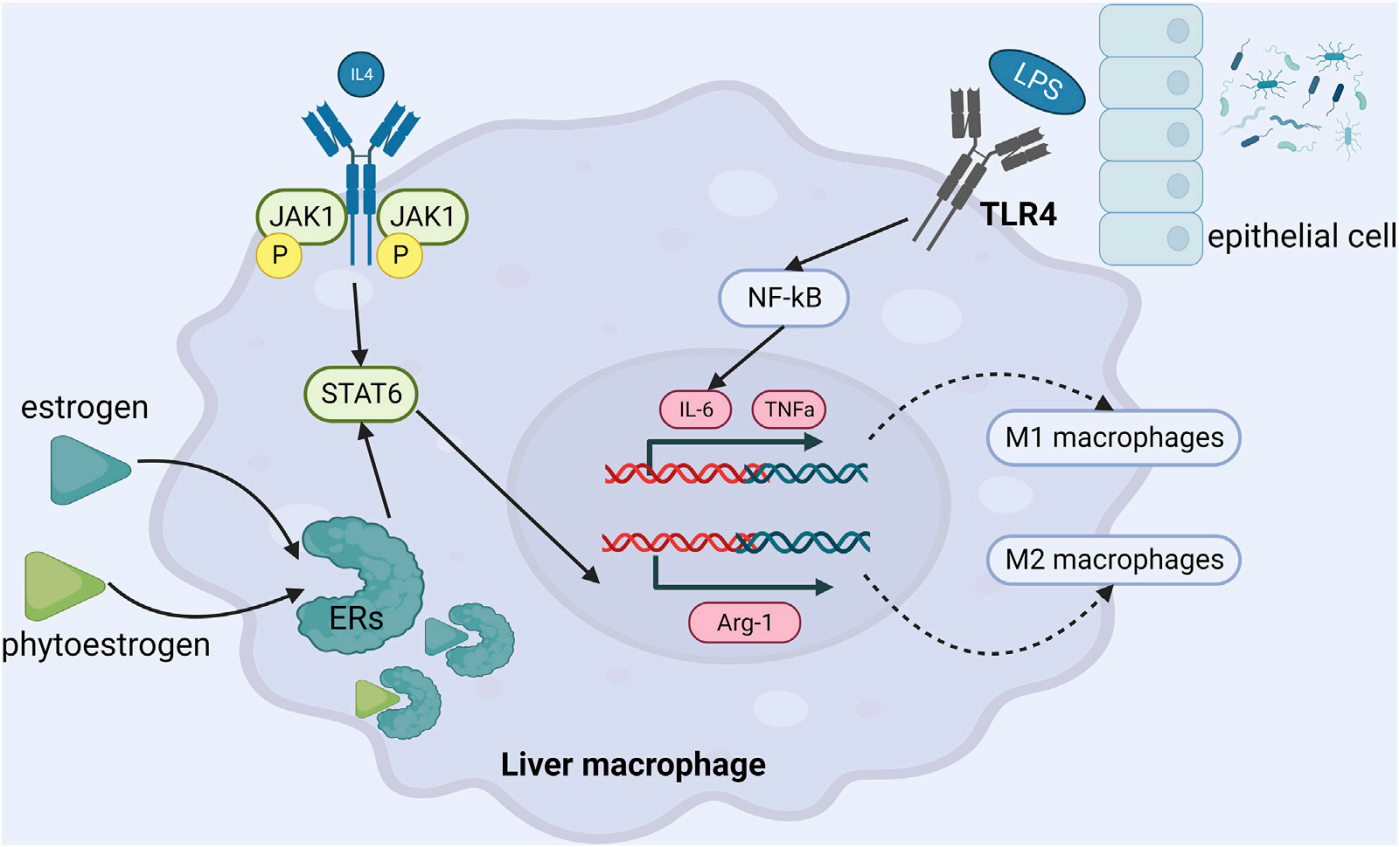
The mechanism of phytoestrogens in alleviating liver inflammatory response.

A study from author Cao YK (Cao et al., 2013) has shown that supplementation of daidzein (50 mg/kg) could reduce serum inflammatory factors in OVX rats, such as TNFα, IL-6. Another study from author Nanashima N (Nanashima et al., 2020) showed that dietary supplementation of 3% Ribes nigrum could reduce TNFα, IL-6 and IL-1β in OVX rats and improve the pathological state of liver inflammation. Similarly, the supplementation of 500 mg/kg flavonoids quercetin (Quercitrin) could increase the serum estrogen level in OVX mice with NAFLD and reduce the expressions of TNFα, IL-6 and IL-1β (Hur et al., 2020). In the studies about the beneficial effects of genistein in the C57BL/6 mouse NASH model, Zamani-GF et al.and Gan M et al.found the anti-inflammatory pathway of genistein. On the one hand, genistein promoted the transformation of macrophages into M2-type anti-inflammatory phenotype, reduced the infiltration of M1-type pro-inflammatory macrophages, thereby reduced the secretion of pro-inflammatory factors and ultimately suppressed expression of NF-κB (Zamani-Garmsiri et al., 2021). On the other hand, genistein directly inhibited the secretion of inflammatory factors and achieved anti-inflammatory effect by up-regulating the expression of miR-451 in liver (Gan et al., 2019). Many other studies have confirmed that genistein exerts anti-inflammatory effects by inhibiting the expression of Toll-like receptor 4 (TLR4) and reducing the levels of downstream TNFα, IL-6, endotoxin and 8—isoprostaglandin (Yalniz et al., 2007; Ji et al., 2011; Incir et al., 2016; Yin et al., 2019). Xu et al. (Xu et al., 2021) found that genistein regulated (CD68 + CD163)/(CD68 + CD206) protein expression through JAK2/STAT3/SOCS3 signaling pathway, thereby regulating the proportion of liver M1/M2 macrophages, reducing the level of IL-1β, IL-6, TNF-α and monocyte chemotactic protein 1 (MCP-1), and playing an anti-inflammatory role. (Figure 3).

### Regulation of intestinal flora

With the development of ‘gut-liver axis’ theory and gene sequencing technology, the role of intestinal flora in the pathogenesis of NAFLD has received extensive attention, which provides a new target for the treatment of NAFLD. Clinical studies (Zhao et al., 2019) have shown that compared with premenopausal women, the ratio of M. intestinalis/*Bacteroides* (F/B) in postmenopausal women increases, and higher F/B is related to obesity. This may be due to decreased estrogen levels in postmenopausal women causing fat redistribution, mainly abdominal fat increase. Therefore, maintaining intestinal microecological balance in postmenopausal women may become a new strategy for the treatment of postmenopausal NAFLD.

There is an interaction between phytoestrogens and intestinal flora. Phytoestrogens are mainly converted into compounds with estrogenic activity by intestinal microorganisms *in vivo*. For example, intestinal microorganisms can convert daidzein and genistein into equol derivatives (Zhao et al., 2019; Á et al., 2020), so the composition of intestinal flora will affect the metabolism of phytoestrogens. On the other hand, studies have confirmed that phytoestrogens and their metabolites can also regulate and reshape the composition of intestinal microorganisms. A study (Naudhani et al., 2021) found that formononetin regulated intestinal microbial balance by increasing the number of *Clostridium* aldenense, Clostridaceae unclassified, cluster true bacteria, acetate and butyrate-producing bacteria, and maintained the integrity of the intestinal membrane by up-regulating the expression of Muc-2 and occludin. Consistent with previous studies, another study (Cardona et al., 2013) found that genistein reduced serum endotoxin levels by selectively increasing Akkermansia muciniphila to alter intestinal microflora in mice, thereby achieving anti-obese effects. In addition, berberine could increase the abundance of beneficial bacteria in OVX rats, such as *Bacteroides*, Bifidobacterium, *Lactobacillus* and Akmania (Fang et al., 2021). Moreover, genistein, daidzein and Humulus lupulus L. extract could improve intestinal mucosal barrier function, increase intestinal microbial diversity and reduce the abundance of pathogenic bacteria (Hamm et al., 2019; Ou et al., 2019; Ortega-Santos et al., 2020). A clinical study (Ou et al., 2019) found that compared with placebo, genistein (50 mg/day) treatment for 2 months could increase the number of intestinal Verrucomicrobia in obese patients, reduce serum endotoxin level and improve IR.

### Anti-hepatic fibrosis

Ko SH’s study found that postmenopausal women had a higher incidence of NAFLD than premenopausal women (Ko and Kim, 2020). Importantly, studies found that the longer estrogen deficiency, the higher the risk of liver fibrosis in postmenopausal NAFLD patients (Klair et al., 2016). A number of studies have found that estrogen can inhibit the activation of hepatic stellate cells and prevent the progression of fibrosis. (Li et al., 2021; Lin et al., 2021; Šisl et al., 2022).

*In vivo* and *in vitro* studies have confirmed that phytoestrogens have antifibrotic effects. Many studies have confirmed that calycosin can improve the C57BL/6 liver fibrosis mouse model induced by carbon tetrachloride (CCL4). Studies by Zhang et al. (Zhang et al., 2021) and Deng et al. (Deng et al., 2018) confirmed that calycosin could improve liver fibrosis in C57BL/6 mice induced by CCL4. Zhang et al.and Deng et al.confirmed that calycosin can improve CCL4-induced liver fibrosis in C57BL/6 mice.

They reported a variety of mechanisms. Firstly, calycosin could increase matrix metalloproteinase-1 (MMP-1) expression, inhibit tissue inhibitors of metalloproteinases-1 (TIMP-1) expression, increase MMP-1/TIMP-1 ratio, inhibit collagen synthesis, and balance MMP-1/TIMP-1 system (Zhang et al., 2021). Secondly, calycosin inhibited fibrosis by increasing ERβ expression and activating JAK2-STAT3 pathway. Thirdly, calycosin significantly inhibited the proliferation and migration of activated hepatic stellate cells (HSCs) (Deng et al., 2018). Ganai et al. (Ganai and Husain, 2017) induced liver fibrosis in rats with d-galactosamine (D-GalN) and supplemented them with genistein (5 mg/kg body weight) for 12 weeks. At the end of the experiment, it was found that genistein could inhibit the accumulation of α smooth muscle actin (αSMA), which is a marker of HSC cell activation. TGF-β/Smad signaling pathway is a star pathway in the process of liver fibrosis. Ganai et al. (Ganai and Husain, 2017) found that genistein could play an anti-fibrosis role by blocking TGF-β/Smad signaling pathway. Xu et al. (Xu et al., 2021) confirmed through *in vivo* experiments that the supplementation of genistein also improved liver fibrosis in rats induced by dimethylnitrosamine (DMN). Genistein could inhibit the expression of αSMA and type I collagen α1 in rat liver and improve liver pathological injury. At the same time, *in vitro* experiments performed with genistein on HSC cell line LX2 cells confirmed that genistein could inhibit the viability and proliferation of LX2 cells, and it was important to induce LX2 cell cycle arrest in G0/G1 phase. Although Ganai et al. and Xu et al. used different methods to induce liver fibrosis, they all clarified the anti-hepatic fibrosis effect of genistein.

## Conclusion and future perspectives

**FIGURE 4:**
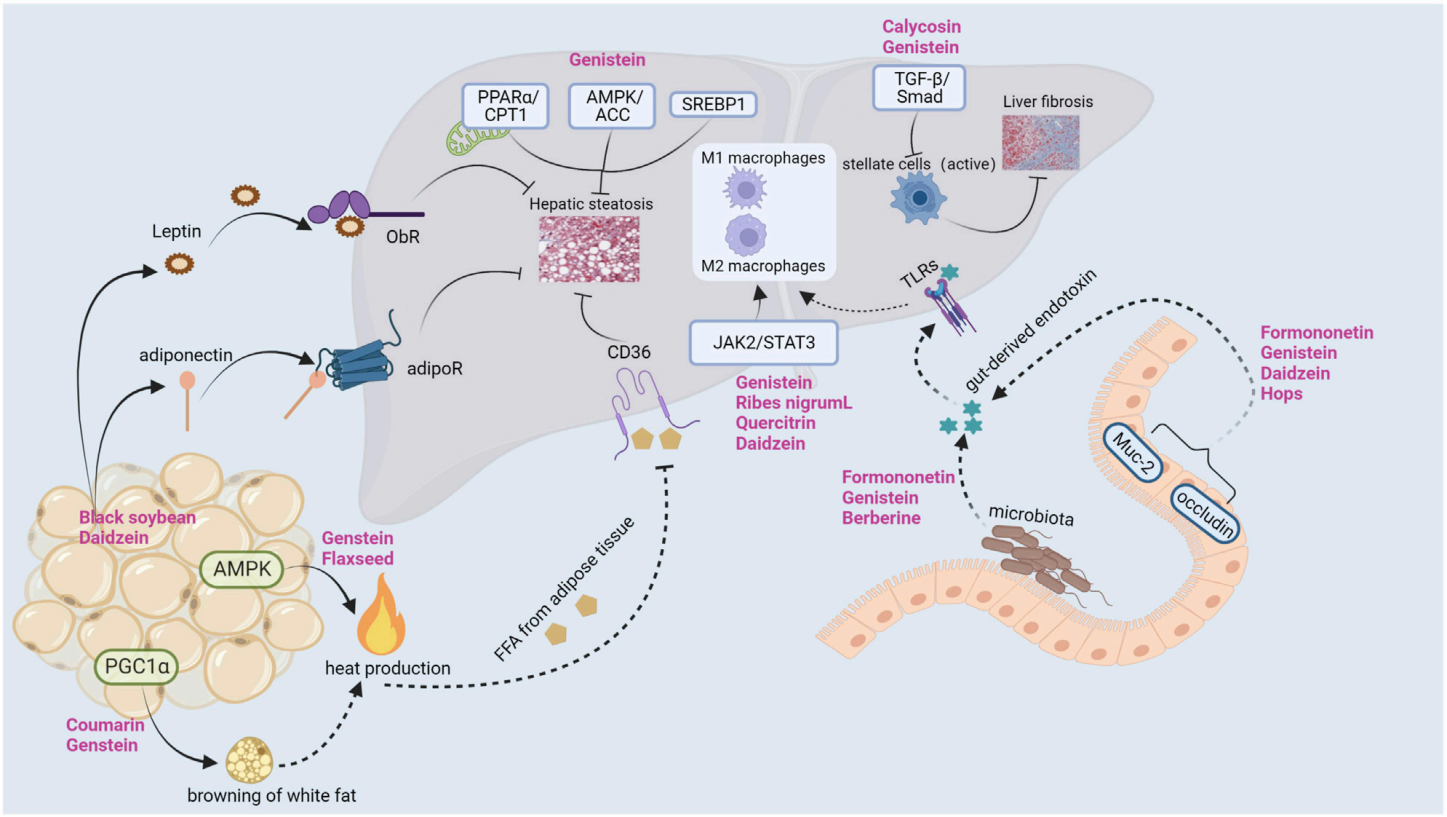
Role of phytoestrogens in postmenopausal NAFLD.

The risk of metabolic diseases such as NAFLD, T2DM, hyperlipidemia, metabolic syndrome, obesity and cardiovascular disease in postmenopausal women has increased significantly (Eghbali-Babadi et al., 2021; Harraqui et al., 2022; Mishra et al., 2022; Saigo et al., 2022; Tang et al., 2022), which has attracted more and more attention from clinical and researchers. Estrogen deficiency may be the main culprit for accelerating blood lipids, glucose metabolic disorders, IR, imbalance of oxidation and antioxidant systems, and intestinal flora imbalance (Ko and Jung, 2021; Liu et al., 2022). However, there is an increasing risk of adverse events in clinical estrogen supplementation, so finding safe and alternative estrogen supplementation drugs is a difficult problem to be solved in clinic. Phytoestrogens are common in diet and can exert many biological effects observed in cells, animals and humans. The research on phytoestrogens has increased dramatically in the past few years, especially in animal and cell experiments. In this paper, we summarize the mechanism of phytoestrogens improving postmenopausal NAFLD through multiple pathways, multiple targets and multiple organs (see Figure 4).

## Conclusion

Although most studies have confirmed that phytoestrogens can improve the metabolic problems of NAFLD in postmenopausal women, such as weight gain, abdominal obesity, elevated blood glucose, and elevated blood pressure. However, there are still many limitations. Currently phytoestrogen-related clinical studies are less than basic studies. There is a lack of observation or evaluation methods for liver histopathology in clinical studies. It is necessary to conduct additional studies to evaluate the long-term efficacy and side effects of phytoestrogens on human beings. The safety of drugs is one of the important concerns in clinical research. The dosage, dosage form and course of treatment of phytoestrogens are not uniform in the reported experiments. It is still necessary to evaluate the beneficial and harmful doses of phytoestrogens to the human body, and the effects of phytoestrogens on other drugs or dietary products. Phytoestrogens and their activities are complex and species-specific. It is still necessary to carry out research to assess the gender differences in human responses to phytoestrogens, so as to better provide clinical reference for postmenopausal NAFLD patients. Although there are many kinds of phytoestrogens, the clinical reports of phytoestrogens are mainly about genistein. Therefore, it is necessary to further explore and study the efficacy of other phytoestrogens on human body, and provide data for clinical research of new drugs.

At present, most of the current research on phytoestrogens focuses on postmenopausal women. What is the effect of phytoestrogens on adult males ? Different studies have come to the controversial conclusion. Rashid Rdeng et al. (Rashid et al., 2022) found that Genistein reduced male testosterone levels, reduced sperm quality, and lowered fertility. In contrast, Reed KE et al. (Reed et al., 2021) found that regardless of dose and study duration, neither soy protein nor isoflavone exposure affects TT, FT, E2 or E1 levels in men. There is a clear need for further carefully designed studies to elucidate the effects of phytoestrogen consumption on adult males.
